# Policy Approaches Toward Combatting Venereal Diseases in the Soviet Occupation Zone in Germany (1945–1949), the German Democratic Republic (1949–1989), and the Polish People's Republic (1945–1989)

**DOI:** 10.3389/fmed.2021.581739

**Published:** 2021-05-28

**Authors:** Marcin Orzechowski, Katarzyna Woniak, Maximilian Schochow, Florian Steger

**Affiliations:** Institute of the History, Philosophy and Ethics of Medicine, Ulm University, Ulm, Germany

**Keywords:** hygiene, public health, medical history 20th century, sexually transimitted diseases, legislation and jurisprudence

## Abstract

The spread of venereal diseases after the Second World War constituted a grave public health danger in Europe. Especially in all four occupation zones in Germany and the Polish People's Republic high morbidity rates were observed. In order to limit the spread of diseases, respective administrations adopted specific regulations. The aim of this research is the analysis and comparison of legal regulations for controlling and combating venereal diseases in these countries. We have analyzed legislative and administrative acts concerning combatting venereal diseases issued by the official organs of the Soviet Occupation Zone, the German Democratic Republic, and the Polish People's Republic from 1945 to 1989. Subsequently, the analyzed sources were evaluated in light of the existing literature on the topic. Our analysis shows that policy approaches in both countries were based the Soviet Union's model for fighting venereal diseases. Visible are similarities of the approaches. They include organization of anti-venereal services, compulsory hospitalization, and actions against social groups perceived as sources of venereal diseases. Beside the purpose of breaking the spread of the epidemics, the approaches had also a political aim of sanctioning behavior that diverged from prescribed socialist moral norms.

## Introduction

After the Second World War, venereal diseases constituted one of the major threats to public health in Europe. According to the data of the World Health Organization presented by King (1), morbidity rates in several European countries, i.e., in Norway, Denmark, Greece, Italy, and France, reached their peak during wartime or shortly after the war. The epidemiological outbreak was influenced by multiple factors, such as deregulation of the norms of social life, movements of troops, the destruction caused by military operations, massive migration of the people, administrative chaos, and general impoverishment of the population (2). In addition, desolate post-war medical infrastructure played a major role. Scarcities in medication, low number of practicing physicians, lack of hospital beds contributed to rising numbers of individuals with venereal diseases (3).

This situation was especially visible in the areas particularly affected by the war: the four occupation zones of Germany and in the Polish Peoples' Republic (PPR) (4–7). In 1945, morbidity rates of syphilis and gonorrhea were alarmingly high. The hazard of spreading venereal diseases constituted a serious problem in the German Democratic Republic (GDR) and in the PPR also in the following decades (3, 8). After the initial successes in containing the epidemic, subsequent waves of gonorrhea ensued in the 1950s and 1960s (9, 10). The epidemiological situation has only been contained in the PPR in the late 1970s (10), whereas the GDR observed high morbidity rates almost until the end of the communist system in this country (9). This severe situation required determined action. Breaking the epidemiological spread of venereal diseases became an important task for the authorities in both GDR and PPR. As a result, several legal acts were issued, which aimed at combatting the challenge of venereal diseases for public health (2, 3, 11).

In recent years, a considerable amount of research was published on the topic of public health measures in combatting the spread of venereal diseases in the GDR (11–14). These works suggest that policy measures toward combatting venereal diseases in this country were influenced by the model adopted from the Soviet Union. As the Soviet Occupation Zone (SOZ), the GDR, and the PPR belonged to the Soviet sphere of domination, the underlying hypothesis is that their policy measures were similar and congruent with the Soviet model. However, a systematic analysis of legal regulations and a precise determination of approaches toward the topic have not yet been conducted. Therefore, the aim of this research is the comparison of legal regulations for the control and combating venereal diseases in the SOZ, the GDR, and the PPR during the communist period (1945–1989). In our examination, we focused on providing answers to the following research questions: (1) Which policy measures were adopted by both countries after 1945? (2) What are the similarities and differences between legal acts in this area in Germany and Poland?

## Materials and Methods

The main sources used for this analysis were legislative and administrative acts concerning the fight against venereal diseases issued by the official organs of the SOZ in Germany, the GDR, and the PPR during the period 1945–1989. For Poland, these legal acts were searched in the electronic database of legislative acts ISAP — the Internet System of Legal Acts. The search was conducted using the following keywords: “venereal disease,” “gonorrhea,” “syphilis,” and “sexually transmitted disease.” The search identified 37 records, of which full-texts were examined for relevance to the research topic and research questions. Out of these 37 records, 5 legislative acts are included in the following analysis. Because corresponding norms for the SOZ and the GDR are not archived electronically, the texts of legal acts issued in the SOZ and the GDR were acquired from secondary literature (15).

In addition, we have reviewed secondary literature on the topic of combating venereal diseases in both countries. Through a search in databases for scientific literature PubMed/MEDLINE and Web of Science, we have identified books and articles discussing policy approaches to the issue of venereal diseases in both countries. The review of the secondary literature served the purpose of investigating practical instruments of implementing the normative policy measures. Based on this review, we have analyzed approaches toward combatting venereal diseases in both countries during the communist period.

## Results

### Legal Framework

Breaking the epidemiological spread of venereal diseases became one of the most important tasks of the authorities in both countries. As a result, several legal acts were issued. The importance of these laws shows the fact that they were among the earliest legal initiatives undertaken by both respective administrations after the war. Table 1 provides an overview of legal acts introduced in both countries and the main regulatory points of these acts.

**Table 1:** Overview of the analyzed regulatory acts regarding combatting venereal diseases in the Soviet Occupation Zone, the German Democratic Republic, and the Polish Peoples' Republic.

**Title of the regulation**	**Date of issue**	**Main regulatory points**
**Soviet Occupation Zone and German Democratic Republic**		
Law on Combatting Sexually Transmitted Diseases	February 18, 1927	•Compulsory medical treatment in a hospital for persons infected or suspected of being infected with a venereal disease •Immediate enforcement to execute the admission
Order No. 25	August 7, 1945	•Organization of a dense network of venereology institutions •Suppression of legal and illegal prostitution •Compulsory hospitalization of individuals with venereal diseases, especially syphilis
Order No. 030	February 12, 1946	•Organization of local centers for prophylactic treatment and examination •Organization of reformatories for compulsory treatment of prostitutes
Order No. 273	December 11, 1947	•Compulsory treatment of all individuals suspected of spreading venereal diseases in closed venereology wards •Penalties for promiscuous individuals •Obligation to report individuals with venereal diseases by name
Regulation on Prevention and Combatting of Venereal Diseases	February 23, 1961	•Three-step procedure of treatment and compulsory hospitalization •Direct compulsory hospitalization of persons strongly suspected of being infected in a closed ward •Prohibition of sexual contacts for persons with venereal diseases
**Polish People's Republic**		
Decree Regarding Combatting Venereal Diseases	April 16, 1946	•Organization of network of anti-venereal centers •Regulation of form of treatment
Circular No. 7/48	January 22, 1948	•Regulation of form of inpatient or outpatient treatment
Circular No. 51/48	July 6, 1948	•Establishment of mobile sanitary columns
Instruction regarding compulsory hospitalization of people with venereal disease	June 21, 1951	•Regulation of compulsory hospitalization of five categories of individuals with venereal diseases
Regulation Concerning Medical Examinations for Detecting Venereal Diseases	September 2, 1964	•Compulsory examination of persons suspected of prostitution •Compulsory examination and treatment of detained alcoholics

After the end of the Second World War, the Weimar “Law on Combatting Sexually Transmitted Diseases” from 1927 was still legally valid in all four occupation zones of Germany (14). In the Federal Republic, the law was in force until 1953 and was then overridden by the “Law on Combating Sexually Transmitted Diseases” of 23rd of July 1953. In addition, local laws on combatting venereal diseases also existed, for example in the federal state Hamburg (4). In contrast, no local legislation on combatting venereal diseases existed in East Germany. In the SOZ, the Weimar Law was in force until 1947. From 1945 to 1947, it was supplemented by two orders of the Soviet Military Administration in Germany (SMAG): “Order No. 25” and “Order No. 030.” The severity of the epidemiological situation led to the promulgation of a further act: the “Order No. 273,” which repealed the Weimar Law of 1927. This act retained its binding power even after the transition of the SOZ into the GDR in 1949. Only in 1961, the GDR's own act, the “Regulation on Prevention and Combatting of Venereal Diseases” was promulgated by the GDR administration. This act remained in force until 1990.

In the PPR, the central legal act concerning combatting venereal diseases was the “Decree Regarding Combatting Venereal Diseases.” This legal act, with changes introduced in 1947 and 1949, was in force until 2002. A number of regulations and instructions further expanded and complemented norms within this act. The main supplement to this act, “Regulation of the Minister of Health and Social Security Concerning Medical Examinations for Detecting Venereal Diseases,” was issued in form of ministerial regulation and remained in force until 2003. Further central regulations to limit the spread of venereal diseases were issued by the Ministry of Health and included: “Circular No. 7/48 1948 regarding combatting venereal diseases,” “Circular No. 51/48 on movable sanitary columns,” “Instruction of the Health Minister regarding compulsory hospitalization of people with venereal disease.”

Analysis of the legal acts shows similarities of policy approaches toward combating venereal diseases adopted in both countries. These similarities include the organization of anti-venereal services, the obligation of reporting ill individuals and contact tracing, compulsory hospitalization, and actions against social groups perceived as sources of venereal diseases. However, differences are also visible. These include compulsory treatment in closed venereology facilities or wards, which is a specific feature of the GDR's system. The PPR's approach, in contrast, put emphasis on widespread examination and education of the population through mobile medical teams.

### Organization of Venereal Prevention, Examination, and Treatment

From the very beginning, legal acts in both countries provided provisions for guarantying access to anti-venereal services on every level of the country's administration. In the SOZ, the SMAG “Order No. 25” called for the organization of a dense network of venereology institutions on district and county levels, such as out-patient clinics, dispensaries, and hospitals. Through regulations of the “Order No. 030” this network should further be supplemented by counseling centers or welfare stations for prophylactic treatment and examination. In addition, this Order called for the organization of reformatories (“Fürsorgeheime”) under police protection for compulsory treatment of venereal diseases. These were to be located in every district of the Zone as well as in the city of Berlin. They were meant to serve as places for obligatory treatment of individuals that refuse voluntary treatment including prostitutes and women, who infected soldiers of the Soviet Army. Individuals referred to these reformatories were to receive work training courses.

In the PPR, the “Decree of 16th April 1946” introduced a hierarchical organizational structure, in which coordination of all anti-venereal actions lied within the competencies of the Ministry of Health and implementation of actions was to be conducted in healthcare institutions on province and county levels. Reformatories similar to these established in the SOZ were not created. In addition, in order to provide common availability of anti-venereal treatment, mobile sanitary columns were established in 1948 through the “Circular No. 51/48.” The tasks of these columns were to conduct mass examinations and treatment in remote places or places particularly threatened with venereal diseases, educate population, information campaigns concerning venereal diseases, and medical training of physicians in local anti-venereal centers.

### Reporting Obligation and Contact Tracing

Regulations in both countries put special emphasis on the obligation to report venereal diseases and on active detection of possible contacts. Individuals infected with venereal diseases were required, under threat of jail or fine, to report to a physician, who was responsible for obtaining information as to the sources of infection and possibly infected contacts. This information was provided for local and regional health administration. In the authority of these organs laid responsibility for tracking possible contacts, their examination, and treatment. In order to fulfill this task, the medical authorities in both countries were to be supported by police officials.

### Compulsory Hospitalization of Patients

In the SOZ, the Orders of the SMAG defined provisions for compulsory hospitalization of individuals with venereal diseases. Already in the early months of the Soviet occupation, the “Order No. 25” provided that all people with infectious stages of syphilis should be compulsorily hospitalized. The same sanction was to be applied to women considered as prostitutes. Based on the regulations of the “Order No. 273”, individuals with STDs that did not follow the instructions of the attending physician, had sexual contacts against the rules of the Order, broke off the hospital treatment or, based on their lifestyle, could be suspected of spreading the venereal diseases, were subjected to compulsory treatment in a closed hospital. The “Regulation of 23rd February 1961” further extended these rules and provided a three-step procedure for referral for treatment. According to this regulation, a person with a venereal disease could be refered for treatment by a physician of free choice or in an outpatient institution. If the patient did not comply with these two steps, he or she should be subject to compulsory hospitalization in a closed venereal facility.

In Poland, on the basis of the “Decree of 16th April 1946,” regional organs of administration were allowed to order the treatment of individuals with venereal diseases in one of four forms: therapy by a physician chosen by the patient; treatment in a medical facility for general healthcare; treatment in a venereology health center chosen by the regional healthcare administration; or treatment in a hospital ward. In case of patient's resistance, administrative compulsory measures could be employed. The decree left to the regional administrative organs the right to provide detailed directives regulating admitting patients for treatment on an inpatient and outpatient basis. Such directives were issued by the Ministry of Health in the “Circular No. 7/48.” According to it, the choice of the form of treatment was within the responsibility of a district physician. The decision about sending a patient to an outpatient or inpatient treatment should be taken with regard to its purposefulness. More detailed “Instruction of 21st June 1951” identified categories of persons with venereal diseases that were subjected to compulsory hospitalization. Among these were: (1) persons that posed a danger of spreading infection because of their poor housing conditions; (2) persons that did not follow treatment recommendations thus extending the period of the treatment; (3) persons for which hospitalization would ensure rapid recovery and return to employment; and (4) persons that required particular examination or methods of treatment.

### Regulations Concerning Prostitution, Sexual Promiscuity, and Alcoholism

Initial legal regulations in both countries specifically targeted prostitutes as sources of venereal diseases and aimed at the isolation and treatment of this group. However, after 1947, the legal regulations avoided the term, instead they made reference to “sexually promiscuous persons” in the SOZ, or “particular groups of the society” in the PPR. Similarly, the GDR “Regulation of 23rd February 1961” did not mention prostitutes as a particular group of concern. It differentiated between “persons suspected of being infected” and persons “strongly suspected of being infected with venereal diseases.” The second group comprised individuals that repeatedly infected others or frequently changed sexual partners. In case of infection with a venereal disease, such person should be compulsorily hospitalized or committed to a closed venereology ward.

In the legal system of the PPR, prostitution as the source of venereal infections was re-introduced through “Regulation of 2nd September 1964.” This law decreed compulsory medical examination for every person suspected of prostitution and arrested by the organs of police. Furthermore, another social group closely associated in the PPR with spreading the venereal diseases were alcoholics. Therefore, specific actions targeting intoxicated individuals as possible sources of venereal diseases were defined in the “Regulation of 2nd September 1964.” Alcoholics detained in a detoxification center more than once in 2 months were to be subjects of compulsory examination for venereal diseases and stationary treatment. Similar regulation against alcoholics was not included in the legal acts of the SOZ or the GDR.

## Discussion

Before the Second World War, two approaches dominated the discussion on combatting venereal diseases (16, 17). The first, so-called epidemiologic-medical approach, was introduced in the Soviet Union. Already in the 1920s, combatting venereal diseases in the Soviet Union became political agenda and ran parallel to the fight against prostitution, which was perceived as a capitalistic relic. The Soviet model concentrated on early detection and treatment of the diseases combined with educational measures among the population (18–20). Its main features were mass examination of the population and compulsory treatment. Toward this end, a dense network of prophylactic institutions was to be established. Great attention was paid to the so-called “expedition” method of examination, in which mobile medical teams were deployed to rural areas with problematic access to health care. Special emphasis was also put on active detection of the contacts combined with health and social education of the population. Organization of the all anti-venereal actions was to be centrally planned and conducted through health institutions of every administrative level of the state. The introduction of this approach in the Soviet Union led to a drastic drop in the occurrence of venereal diseases (19).

The second approach, the so-called police-sanitary approach, was dominant in several European countries before 1939, among them pre-war Germany and Poland (17, 21, 22). This model focused mostly on policing actions against disease's focal points with emphasis put on actions against individuals and groups that were considered the main sources of spreading the disease. The system mainly concentrated on the fight against prostitution; however, it also aimed at other possible points of origin of infections — alcoholics or sexually promiscuous individuals. Special reformatories and welfare stations were established for these groups, which were perceived as a high threat. This model lacked coordination of activities on a country level and instead mostly concentrated on specified actions within limited areas, such as major cities.

Our analysis of legal regulations in the SOZ, the GDR, and the PPR clearly shows a strong reflection of the Soviet regulations toward venereal diseases. Visible are also distinctions between Germany and Poland. Above all, the analysis shows a more lenient attitude toward compulsory hospitalization of individuals with venereal diseases in Poland.

Political developments in the SOZ, the GDR, and PPR after the Second World War contributed to the formation of the anti-venereal medical service that was similar to the Soviet model. The central prerequisite of this model was the common availability of anti-venereal treatment. In both countries, inpatient institutions were created on the district level and first-contact medical institutions for outpatients such as dispensaries and polyclinics were created on a county level (6, 23, 24). These provided a dense network of healthcare institutions facilitating free-of-charge treatment for people with venereal diseases. In addition, the PPR adopted the Soviet method of “expedition” teams that provided prophylaxis and examination for a wide spectrum of the population (2, 17). Mobile sanitation commissions examined blood across all strata of the population in order to identify the sources of infection. For example, in Poznan, 150.000 examinations were carried out during such actions and several cases with latent syphilis infection were identified (25). Similar mass-scale actions were not conducted in the SOZ or the GDR.

Another similarity of both approaches with the Soviet model was the tracing of infection sources. In both countries, this task laid in the responsibility of the first-contact physicians reporting their findings to the health authorities. The importance of this point is highlighted by the fact that the obligation for contact tracing is repeatedly mentioned in almost all legal acts as well as in the literature on the topic from the period (6, 7, 10). In the PPR, specially trained nurses collected information about the sexual contacts of the infected individuals. The reporting obligation served not only as a source of information on the possible origins of the epidemics but also provided a statistical basis for centrally organized, countrywide actions. Especially these statistical surveys of the population were one of the main aspects of the Soviet health care model (26).

Compulsory hospitalization as foreseen by the Soviet model was applied in both countries. Differences were visible only in the prescribed procedures of compulsory treatment. In the GDR, the law foresaw a three-step procedure in which a patient could be consecutively referred for compulsory examination, stationary treatment, and detention in a closed ward. The procedure built on the top of each step and therefore hospitalization or detention was only the last step of the procedure (11). Closed venereology wards for compulsory treatment were established in all districts of the GDR. A similar three-step procedure did not exist in Polish law. Regulations issued by the Ministry of Health provided the district physician with options as to the relevant treatment procedure. In his decision, the physician could only orient himself by his own assessment of appropriate measures. Compulsory hospitalization was only prescribed for individuals that constituted a threat for spreading the epidemics. There is no information about the existence of closed venereology wards in the PPR similar to those in the DDR. Compulsory treatment of individuals was conducted in hospital venereology wards; however, it took only place in exceptional cases (27).

Given a wider perspective, legal regulations of compulsory commitment to an anti-venereal facility were not exceptional in both countries. Compulsory hospitalization for persons suspected of being infected with venereal diseases was also provided by the “Law on combatting sexually transmitted diseases” implemented on 23rd July 1953 in the Federal Republic of Germany (FRG). It stated that for the purpose of preventing, detecting, and healing sexually transmitted diseases, the fundamental rights to physical integrity and freedom of the individual may be restricted. According to it, admission to a hospital could be enforced in particular cases. Also local legal norms in some German federal states. “Law on Combatting Sexually Transmitted Diseases” promulgated by the federal state Hamburg in 1949, similarly allowed compulsory commitment in certain situations (4).

In practice, compulsory hospitalization in the GDR and the PPR occurred with violation of the legal regulations. In the GDR, girls and women were repeatedly committed to closed venereology stations with the omission of the three-step procedure (11, 13). In many cases, female patients were brought directly to such stations and remained there for several weeks. In these wards, compulsorily committed patients were subjected to daily “work therapies” and political propaganda to become “socialist personalities” (28). “Work therapies” mainly consisted of cleaning chores in the venereology ward and in the hospital. Some contemporary witnesses reported that they were assigned to do the laundry, help in the hospital kitchen, and to clean the toilets. In some cases women were assigned to cleaning duties outside the hospital, or had to work for outside companies, for example as seamstresses in dressmaker factories (11, 14, 28). Such educational function was modeled after the Soviet approach (29). Referral to venereology wards in the PPR also suggests a breach of legal procedures (30). However, instances of “work therapies” or “reeducation” measures similar to those conducted in the GDR are unknown.

The issue of the control of venereal diseases in both countries was closely related to perceived moral problems of the socialist society: prostitution, sexual promiscuity, and alcoholism (8, 31). On the ideological level, these phenomena were associated with “asocial behavior,” unbecoming of a member of a socialist society. In this respect, regulations in both countries provided the legal basis for targeted actions against specific “asocial” groups. The Polish “Regulation of 2nd September 1964” constitutes a clear example of these policies. As an answer to the increased number of infections with gonorrhea at the beginning of the 1960s, the Polish Ministry of Health introduced legislation specifically targeting particular groups: prostitutes and alcoholics. Similar action was not an object of legislation in the SOZ or GDR; however, regulations targeting sexually promiscuous persons provided the legal basis for actions against individuals behaving in a way that was perceived as unwanted in a socialist society. The particular approach in both countries toward the specific social groups deemed as “asocial” is corroborated by the data on compulsory commitment and examination. In 1968, only 774 of the 2.763 (28%) girls and women committed to closed wards in the GDR were diagnosed with and treated for venereal diseases (11). In the PPR in 1977, 70,000 women suspected of prostitution were compulsory tested in 18 venereology clinics. Only 5.150 of these women (7%) were diagnosed with a venereal disease (10).

Considering the effectiveness of the anti-venereal measures, actions taken immediately after the Second World War led to a drastic drop in prevalence of venereal diseases in both countries, especially with regard to syphilis infections (8, 9). In the case of gonorrhea, the effects of regulations introduced in the 1960s in the GDR and the PPR were initially not apparent. A drop in the number of gonorrhea cases was only observable in the PPR starting in the mid of the 1970s (10), whereas in the GDR high rates of infection continued until the late 1980s (9). Lack of visible epidemiological effects indicates that actions undertaken on the basis of the legal acts from the 1960s, especially in the GDR, aimed at sanctioning the behavior of groups not adhering to the socialistic system of values than at breaking the spread of disease.

The results of this research need to be considered in light of its limitations. The aim of is to provide information about the scope of legal acts targeting venereal diseases. Although they give information about the policy approach to the issue, the analyzed legal norms do not reflect everyday practice in the countries under investigation. Especially for the PPR, a detailed analysis of the commitment practice and function, structure, and daily routine of venereology wards should be an object of further investigation. This could provide detailed information on practical implementation of the analyzed policy framework.

The analysis of the policy approaches toward control and combatting venereal diseases in the GDR and in the PPR clearly shows similarities of both systems. Both models followed the Soviet Union's approach. In this regard, policy regulations adhered to the Soviet method of the central organization of mass anti-venereal campaigns, common availability of access to treatment, report and treatment obligation, and compulsory hospitalization of people with STDs. Moreover, visible is that these approaches in East Germany and the PPR had a further aim, besides breaking the spread of the epidemics – to sanction behavior that diverged from prescribed socialist moral norms. Compulsory examination and hospitalization became instruments toward controlling unwanted behavior and thus exemplification of politicization of medicine. Especially in the SOZ and the GDR, the system of compulsory hospitalization served the purpose of re-education of patients in accordance with socialist standards. A more lenient attitude toward compulsory hospitalization distinguished the Polish approach from the one established in the SOZ and the GDR.

## Data Availability Statement

Publicly available datasets were analyzed in this study. This data can be found here: Data is available from the portal Internetowy Spis Aktow Prawnych (ISAP) https://isap.sejm.gov.pl/ and from the secondary literature listed in the references.

## Author Contributions

All authors listed have made a substantial, direct and intellectual contribution to the work, and approved it for publication.

## Conflict of Interest

The authors declare that the research was conducted in the absence of any commercial or financial relationships that could be construed as a potential conflict of interest.
